# Correspondence

**Published:** 1862-11

**Authors:** Charles Nichell

**Affiliations:** Medical Student; Camp Belger, near Baltimore


### CORRESPONDENCE.

Camp Belger, near Baltimore,
September 27, 1862.

Dear Doctor:—I give you the particulars of an interesting though sad affair. This morning at about 12½ o'clock, just as I had returned from the tent of Capts. Gardner and Francis, a corporal called me, stating that a man was stabbed, but when I arrived at the place I found him dead. The particulars are the following: A man named Storey, from Williamsville, belonging to Capt. Kinney's company, attempted to run the guard, (returning to the camp, having previously run it,) the sentinel, Robert Wood, from Evans, belonging to Company A, Capt. Ayres, hailed him, but he did not stop, and ran against the projected bayonet three times, receiving one wound in the left arm, piercing it to the humerus, a second on the crest of the left illium, and a fatal wound through the heart. The bayonet entered at the fourth rib, shattering it about an inch behind the insertion of the cartilage, passing through the left lung and piercing the left ventricle of the heart from below upwards, impinging somewhat upon the structures behind the heart. After he had received these wounds he managed to escape the guard and ran about fifty paces into camp, where he laid down in one of the tents, from which he was carried to the guard house where he died, ten minutes after the last wound.

The most remarkable facts presented in this case, are his continued efforts to pass the guard, and his running that distance while yet his heart had been completely pierced by a large bayonet. Perhaps some of the readers of the Journal may be interested in the facts, and be able to explain this remarkable feature of the case.

Charles Nichell, (Medical Student.)

